# Supplementary figures and images for: Chromatin accessibility, not 5mC methylation covaries with partial dosage compensation in crows
Source: PLoS Genet. 2023 Sep 25;19(9):e1010901. doi: 10.1371/journal.pgen.1010901 (PMC10575545; doi:10.1371/journal.pgen.1010901)

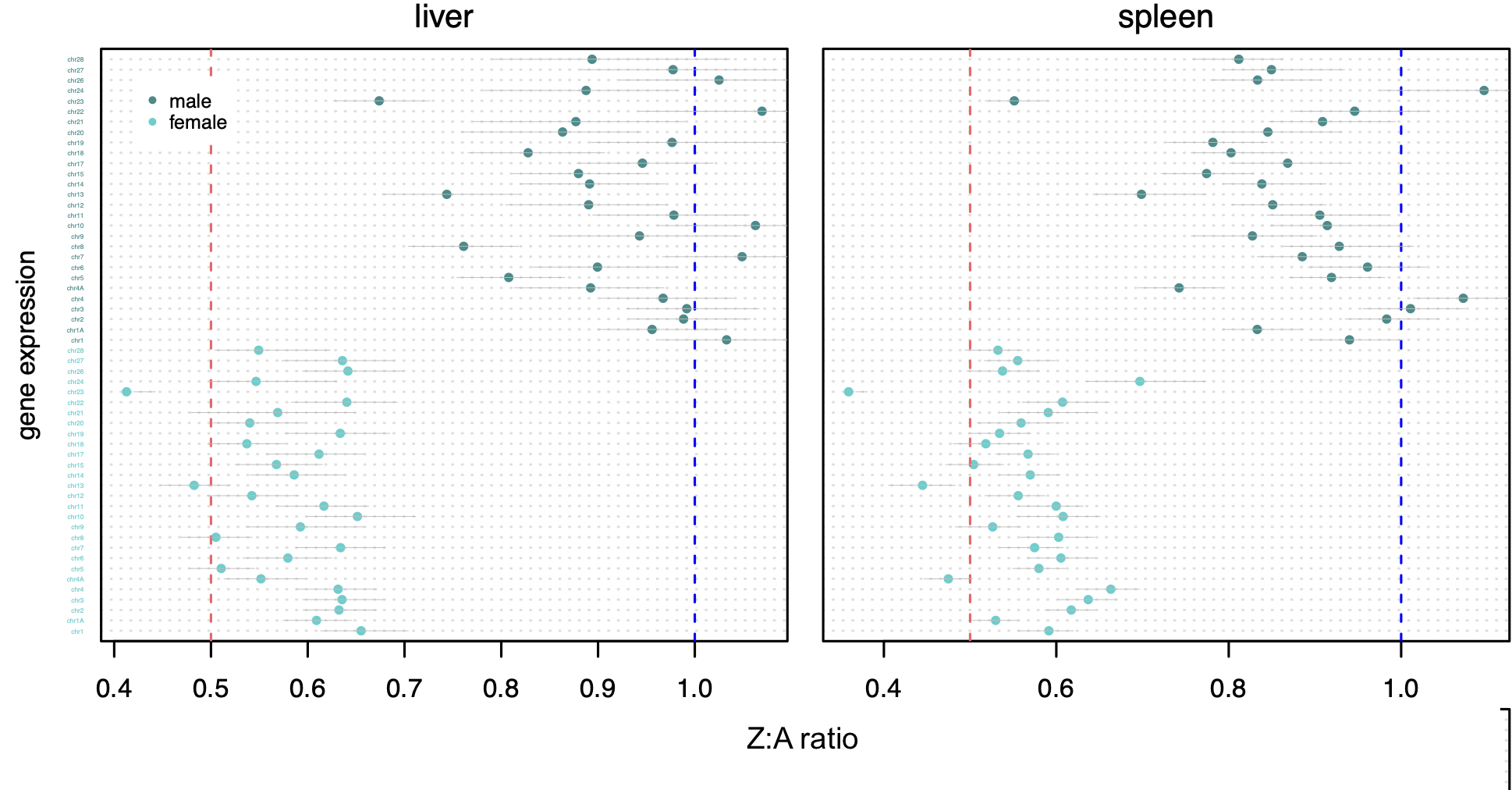

Supplement: S1 Fig — Confidence intervals (95%) drawn from 10,000 bootstraps. Vertical dashed lines show the values for no dosage compensation (0.5) or full dosage compensation (1). (TIFF) [file pgen.1010901.s001.tiff]

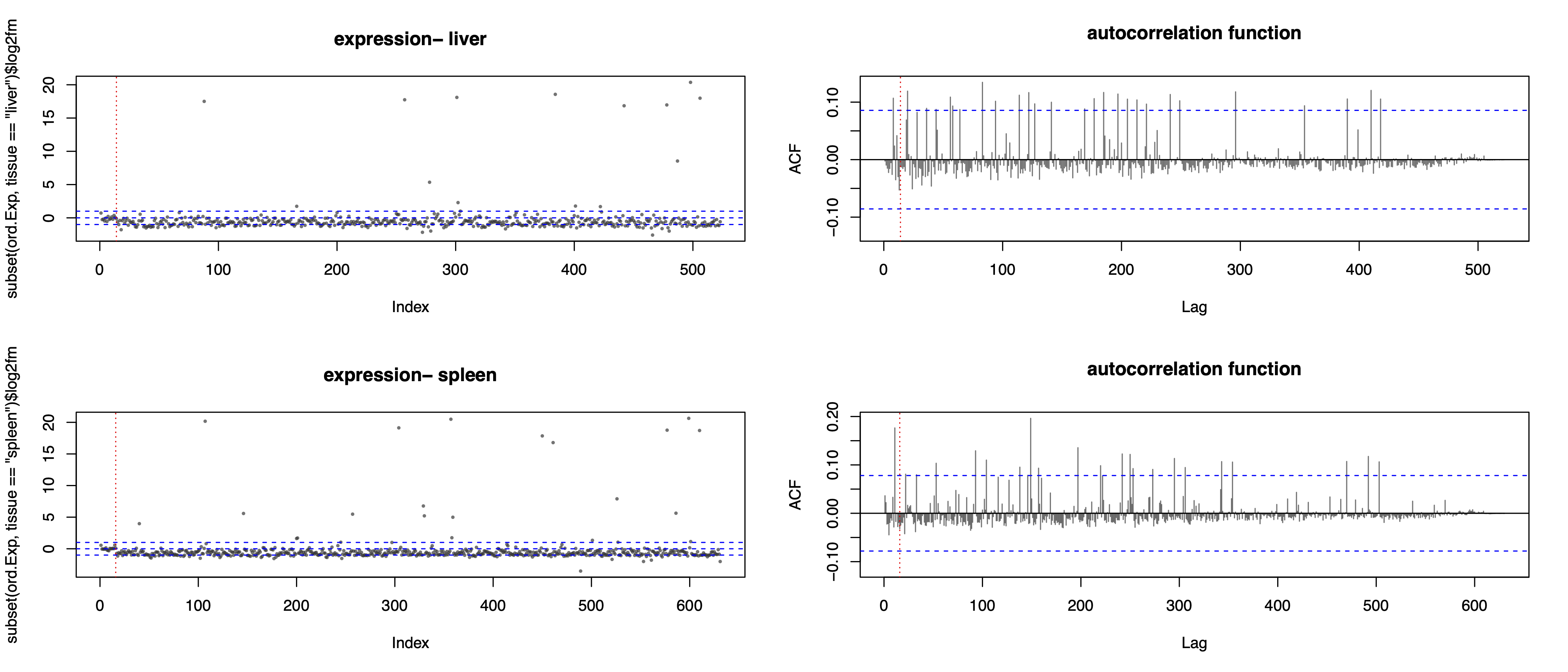

Supplement: S2 Fig — Red vertical line shows the PAR border. Blue lines represent autocorrelation values significantly different from zero. Except for the PAR region, there is no clear trend of clusters that would point at local centers of dosage compensation. Liver: upper panel. Spleen: lower panel. (TIFF) [file pgen.1010901.s002.tiff]

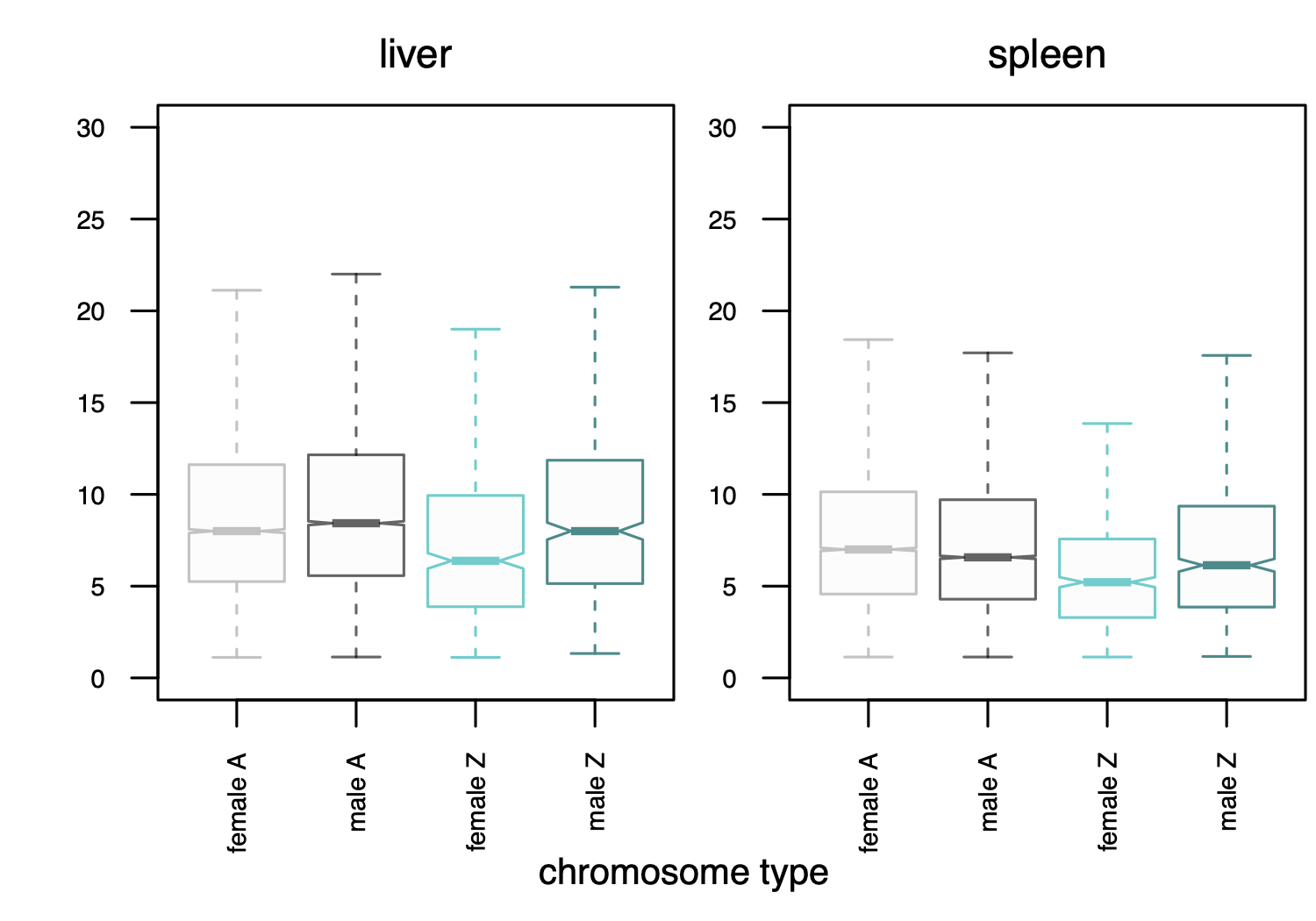

Supplement: S3 Fig — (TIFF) [file pgen.1010901.s003.tiff]

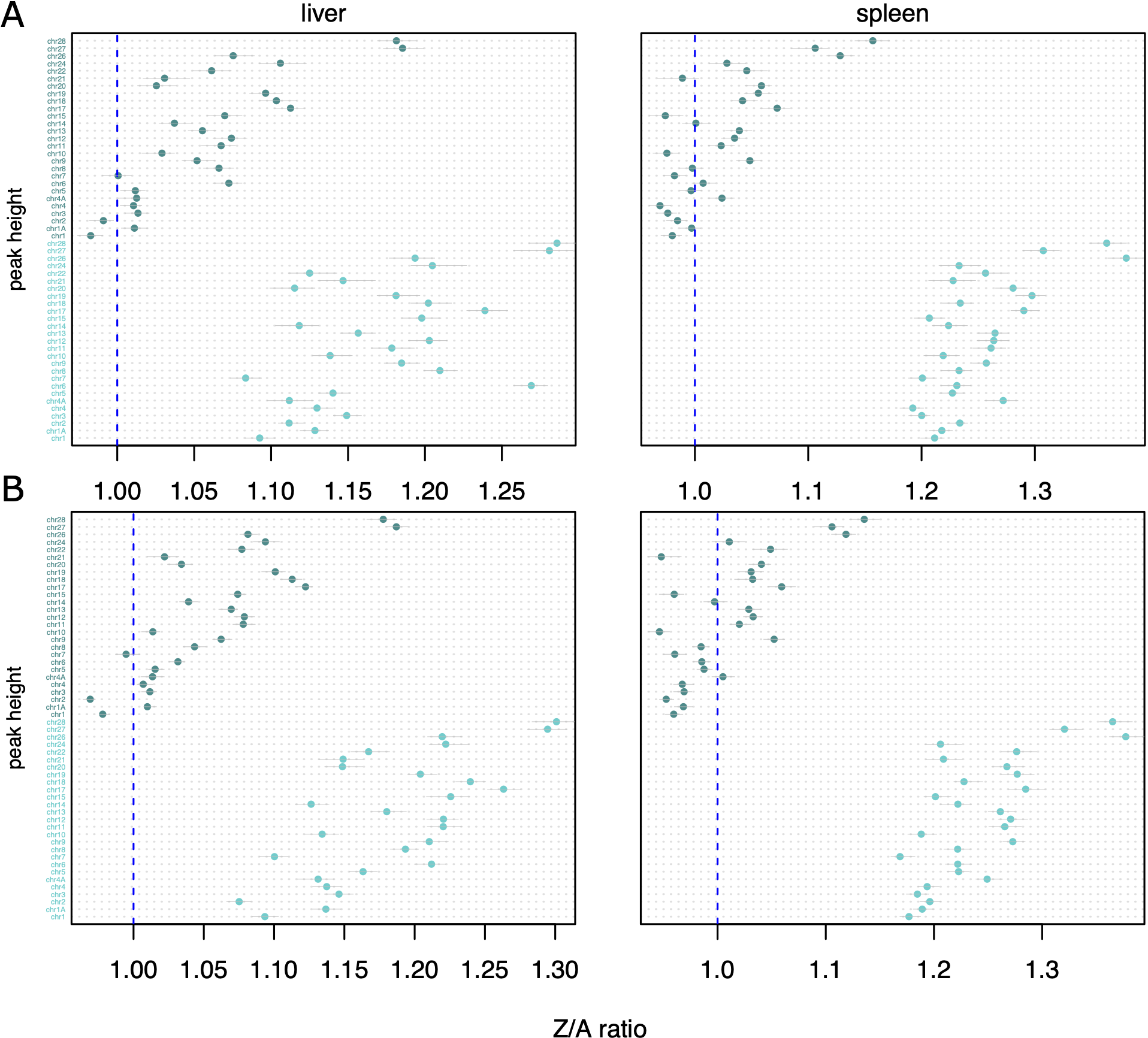

Supplement: S4 Fig — Confidence intervals (95%) drawn from 10,000 bootstraps. (A) shows gene-centered Z:A ratios. (B) shows Z:A only in up- and down-stream regions of expressed genes excluding the gene body. (TIFF) [file pgen.1010901.s004.tiff]

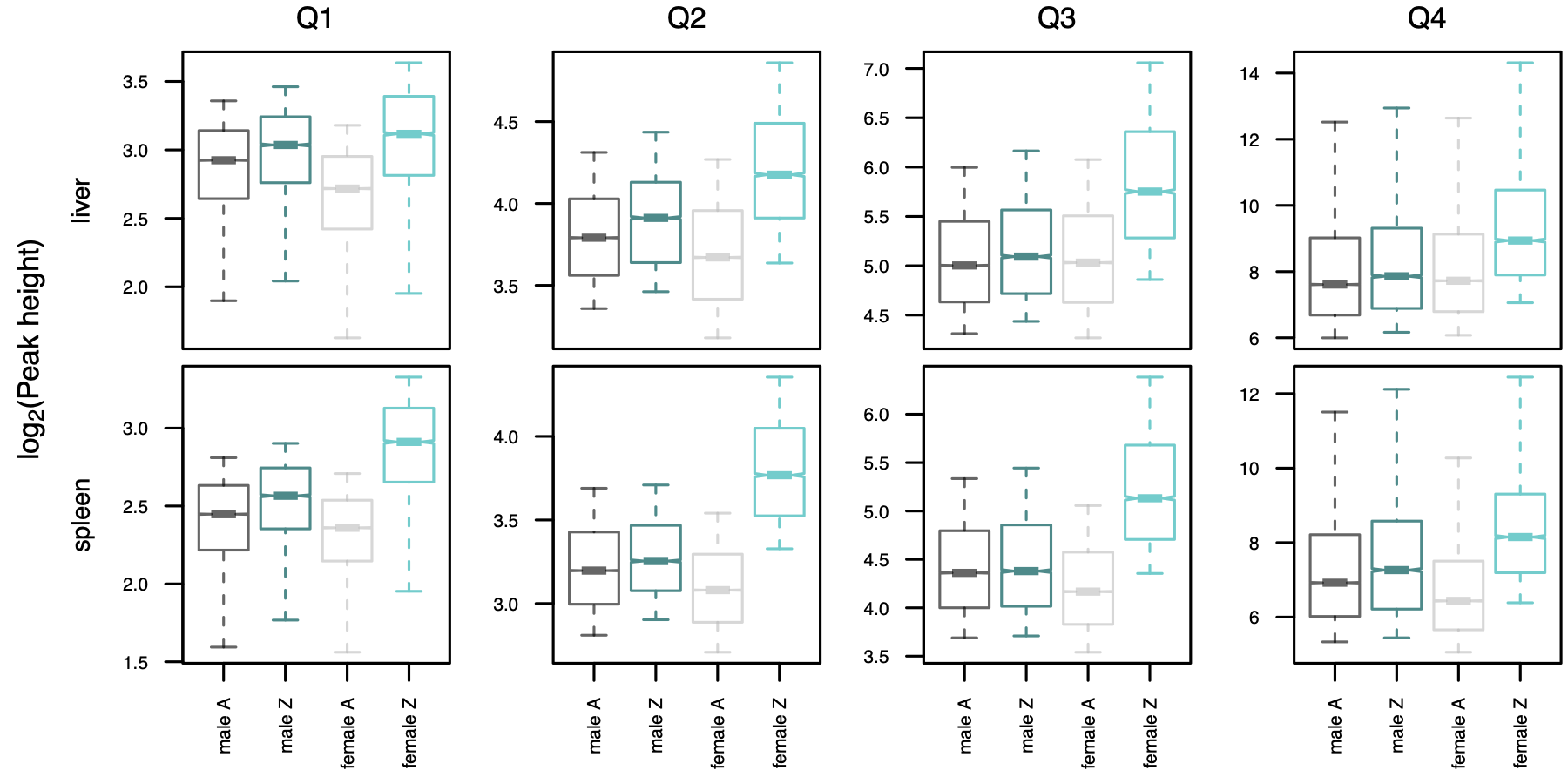

Supplement: S5 Fig — A: autosomes, Z: Z chromosome. Upper panel liver, bottom panel spleen. (TIFF) [file pgen.1010901.s005.tiff]

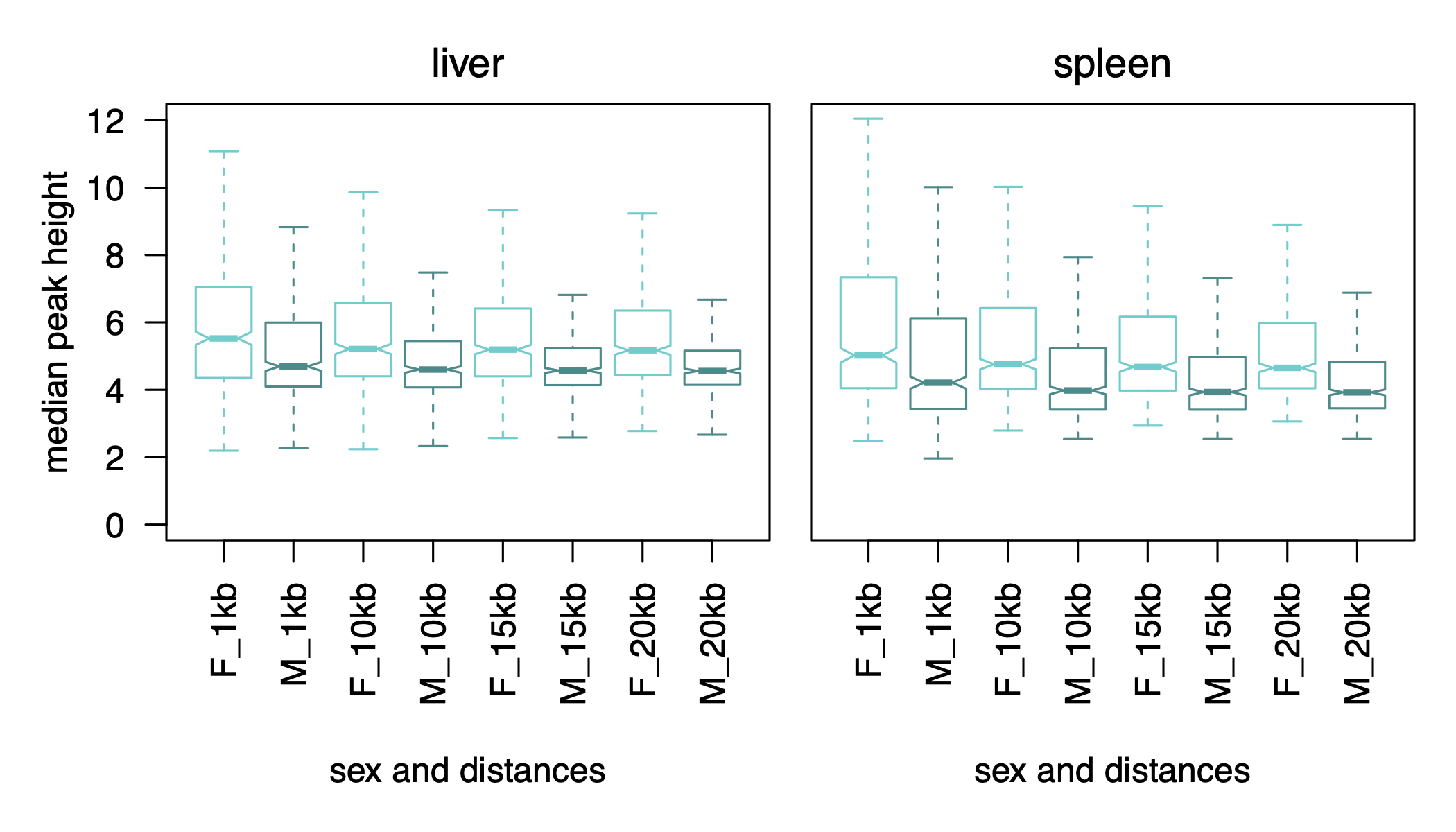

Supplement: S6 Fig — (TIFF) [file pgen.1010901.s006.tiff]

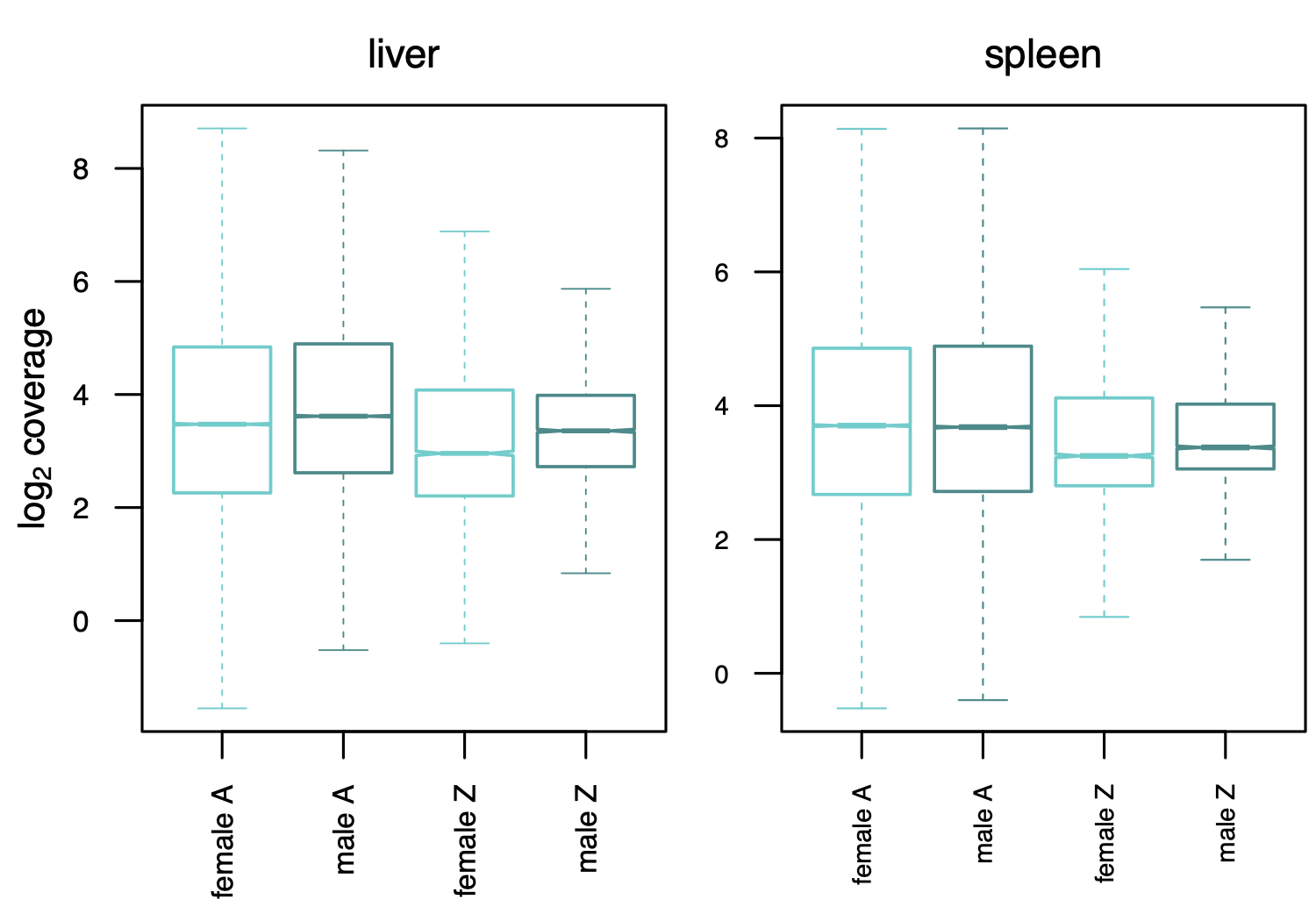

Supplement: S7 Fig — Coverage was calculated at intervals of 100kb. Liver: AAf:AAm = 0.90, Zf:ZZm = 0.76, Zf:AAf = 0.70, ZZm:AAm = 0.84. Spleen: AAf:AAm = 1.02, Zf:ZZm = 0.92, Zf:AAf = 0.73, ZZm:AAm = 0.81. (TIFF) [file pgen.1010901.s007.tiff]

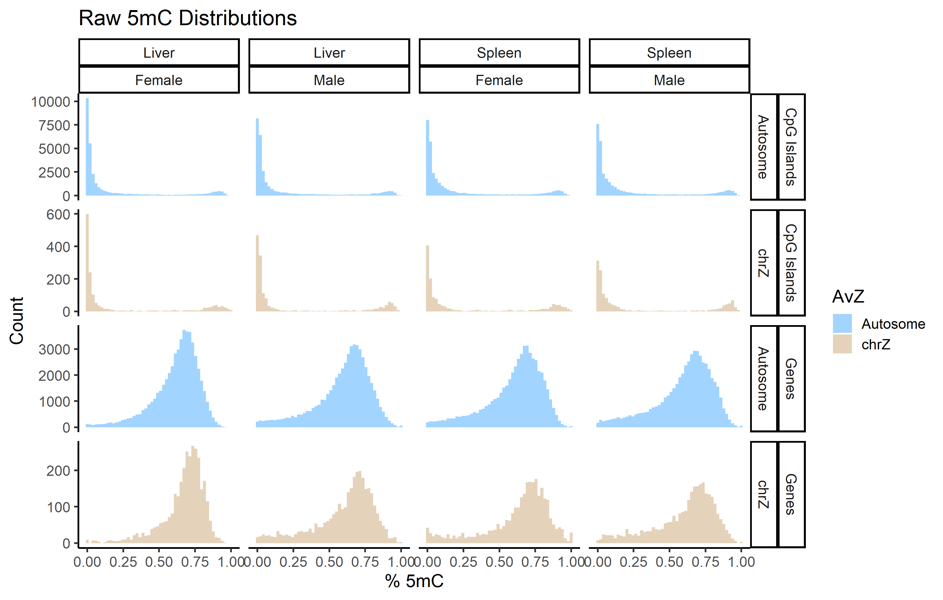

Supplement: S8 Fig — (TIFF) [file pgen.1010901.s008.tiff]

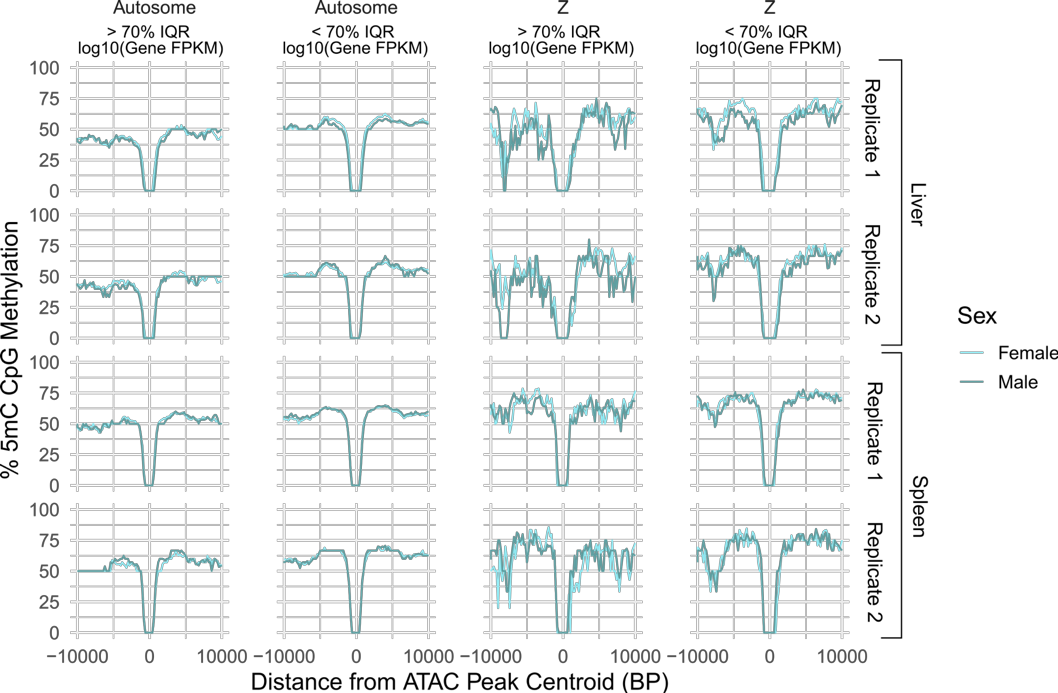

Supplement: S9 Fig — For each sample (n = 8), median methylation levels were calculated in the +/- 10-KB region surrounding a sample’s specific ATAC-seq peak. This 20-kb flanking region was divided into 100 running windows of 200-bp, and the median percent methylation value of each tile was assigned. Before averaging, peaks were divided into more-expressed or less-expressed delimitations based on the log10(FPKM) values of their associated genes, delineated by either peaks above or below the 30% interquartile range of gene expression values, calculated independently for autosomal and Z chromosome genes at the sample-specific level. (TIFF) [file pgen.1010901.s009.tiff]

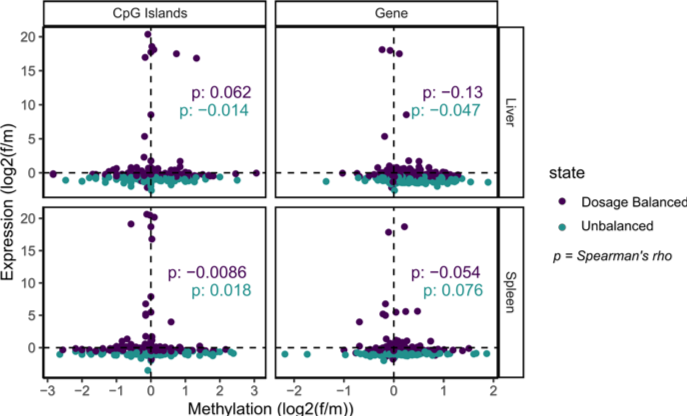

Supplement: S10 Fig — (log2(F/M)) and expression (log2(F/M)) across both tissues for both CpG islands and directly over gene bodies for Z-linked transcripts. CpG island expression level was determined as the expression level of the closest genomic transcript. Spearman’s rho is written for each facet, and for each dosage compensation state (dosage balanced, or unbalanced, as identified from expression data). (TIFF) [file pgen.1010901.s010.tiff]

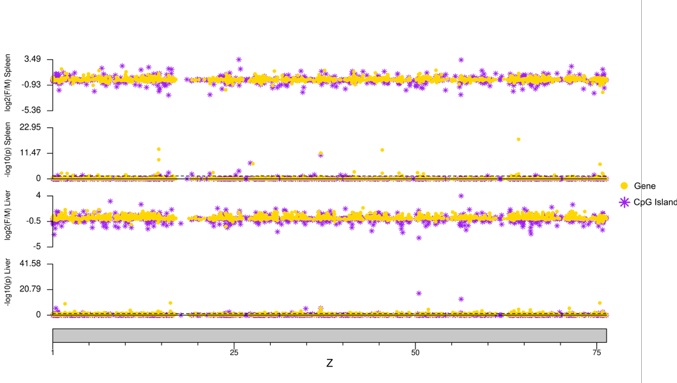

Supplement: S11 Fig — Differentially methylated features (genes or CpG islands) were identified with a beta-binomial regression using methylation counts implemented with a general experimental design framework in DSS. FDR-corrected -log10(p-values) for sex divergence are plotted along the bottom first and third panels for liver and spleen, respectively, with the dashed line indicating FDR-corrected significance (0.05). Bootstrap sampling log2(F/M) values are shown along bottom second and fourth panels. (TIFF) [file pgen.1010901.s011.tiff]

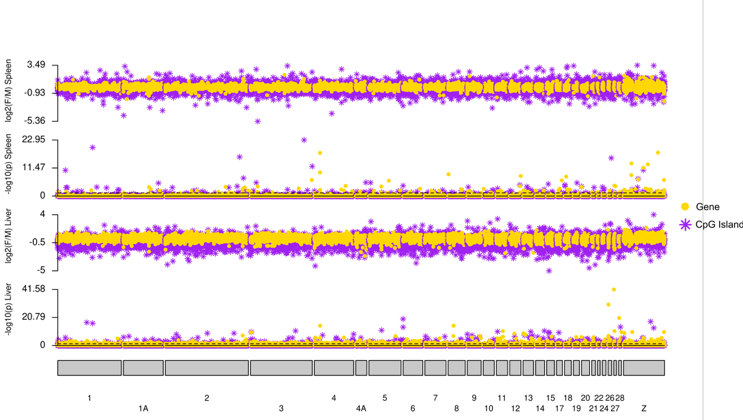

Supplement: S12 Fig — Differentially methylated features (genes or CpG islands) were identified with a beta-binomial regression using methylation counts implemented with a general experimental design framework in DSS. FDR-corrected -log10(p-values) for sex divergence are plotted along the bottom first and third panels for liver and spleen, respectively, with the dashed line indicating FDR-corrected significance (0.05). Bootstrap sampling log2(F/M) values are shown along bottom second and fourth panels. (TIFF) [file pgen.1010901.s012.tiff]

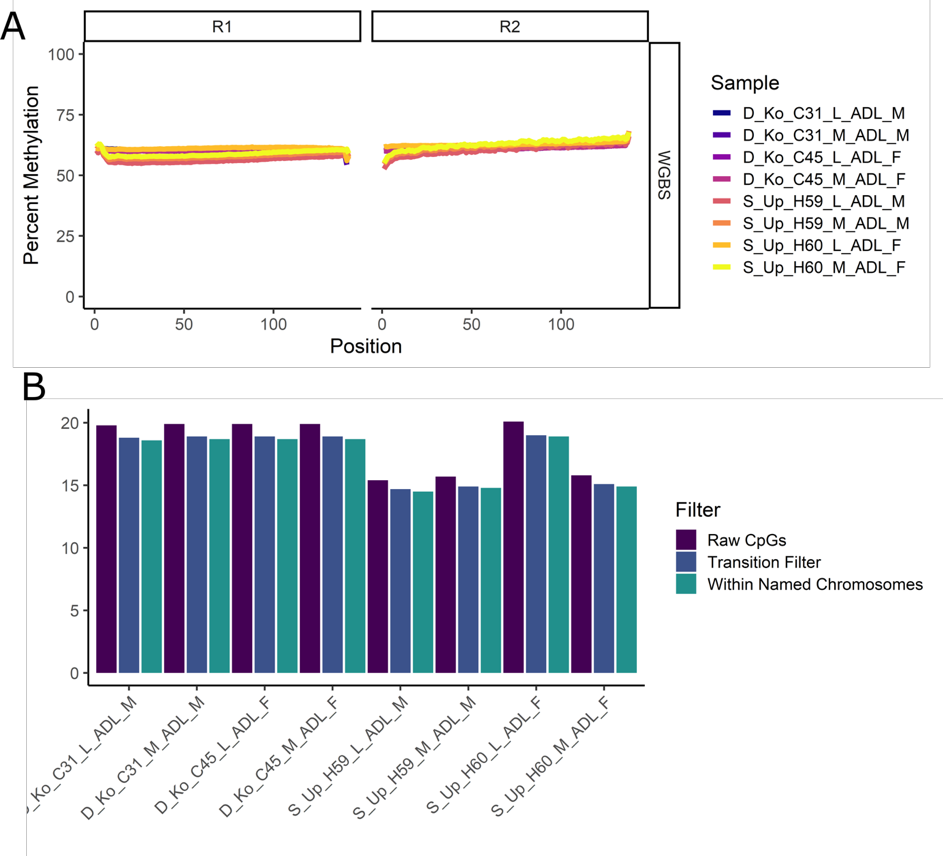

Supplement: S13 Fig — Panel A indicates methylation levels averaged across all reads in a library along read position, used to identify systematic methylation biases along read lengths. Sample name: Individual: S_Up_H59/H60; tissue:liver (L) or spleen (M); age: adult (ADL); sex: male (M) or females (F)). Panel B indicates total 5mC positions called for each sample, as well as sites retained after filtering for C-T and G-A transition SNPs, and retaining sites on assembled chromosomes. (TIFF) [file pgen.1010901.s013.tiff]

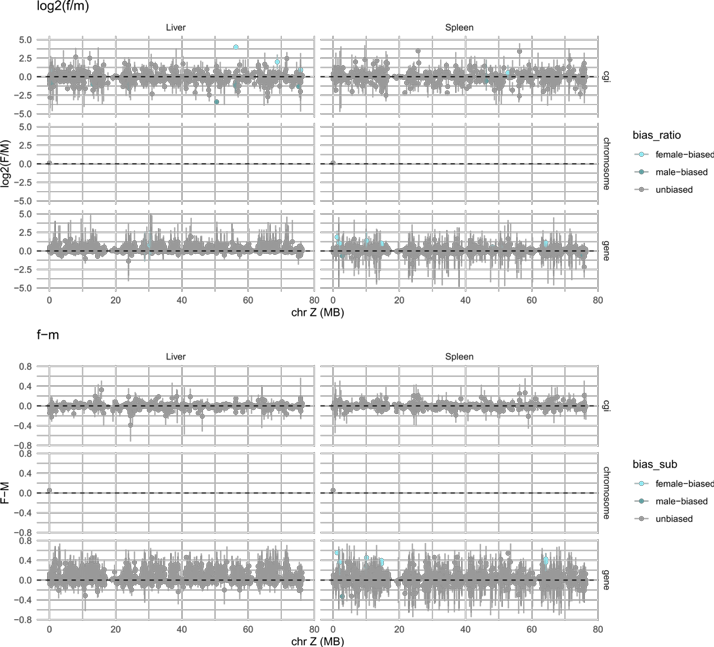

Supplement: S14 Fig — Features were classified as female- or male-biased if they were significantly different for sex from a beta-binomial regression on read counts, and showed either a positive log2(f/m) or f-m greater than 25% (female-biased), or a negative log2(f:m) and f-m less than -25% (male-biased). This analysis also retained female-specific 5mC calls on the Z chromosome which contained 5 reads, thereby providing a lower coverage threshold for the single-copy female chromosome. No differences between these results and the primary manuscript indicate sensitivity issues with technical filtering or analytical parameters. (TIFF) [file pgen.1010901.s014.tiff]

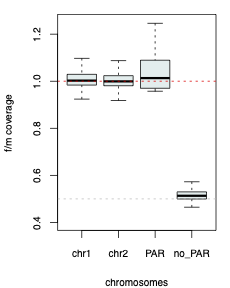

Supplement: S15 Fig — F/M coverage ratio on the shown autosomes and the PAR lie near a ratio of 1 (red line), whereas the hemizygous part of the Z lies close to a ratio of 0.5 (grey line). (TIFF) [file pgen.1010901.s015.tiff]

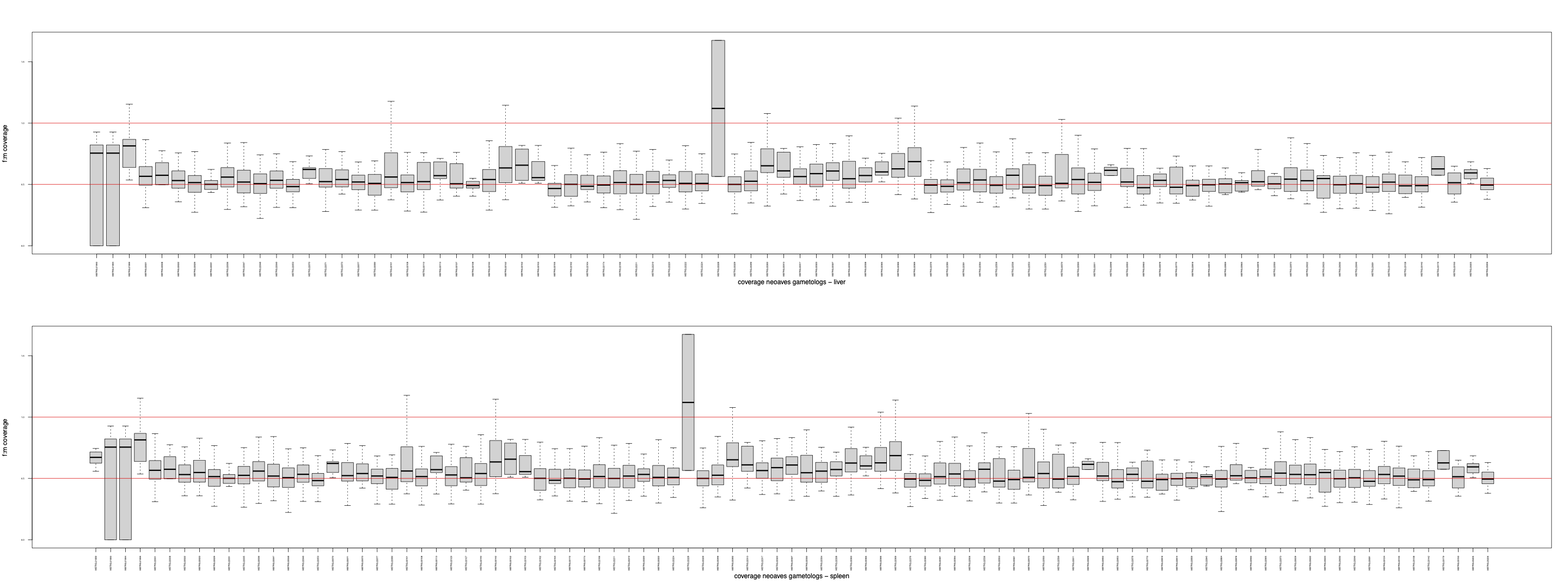

Supplement: S16 Fig — Red horizontal lines mark f:m = 1 and f:m = 0.5. Upper panel: data shown for liver. Lower panel: data shown for spleen. (TIFF) [file pgen.1010901.s016.tiff]

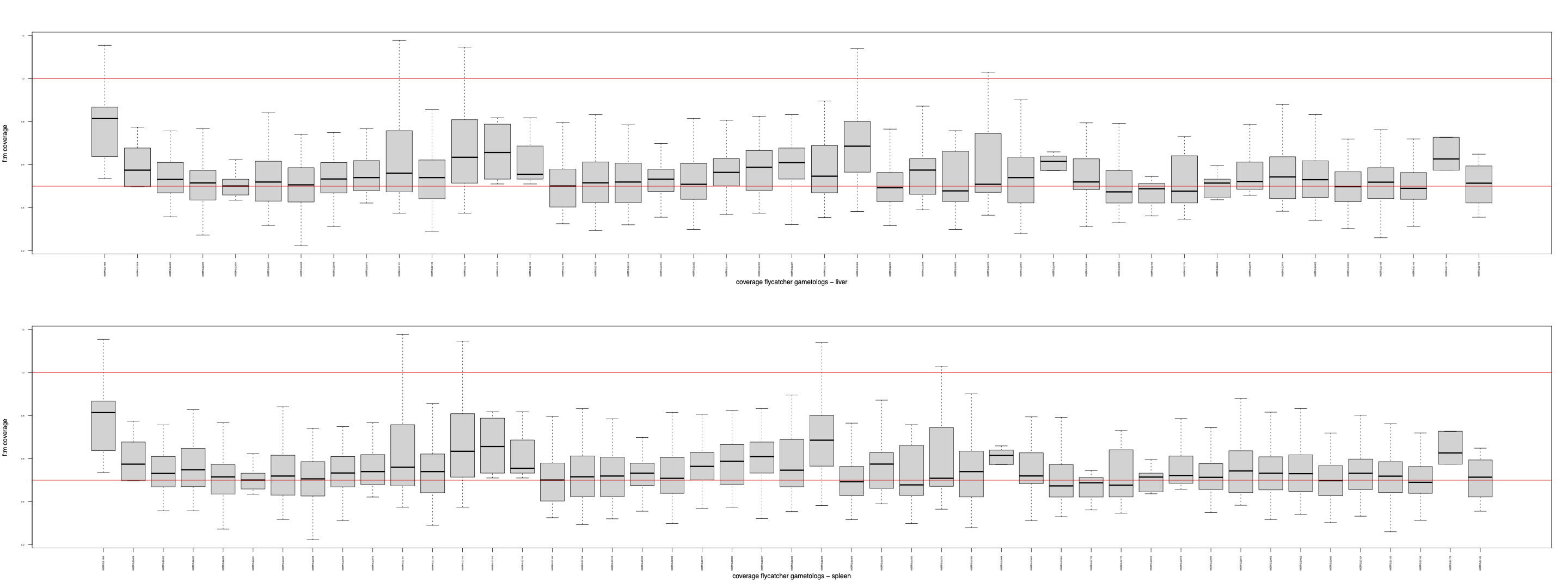

Supplement: S17 Fig — Red horizontal lines mark f:m = 1 and f:m = 0.5. Upper panel: data shown for liver. Lower panel: data shown for spleen. (TIFF) [file pgen.1010901.s017.tiff]

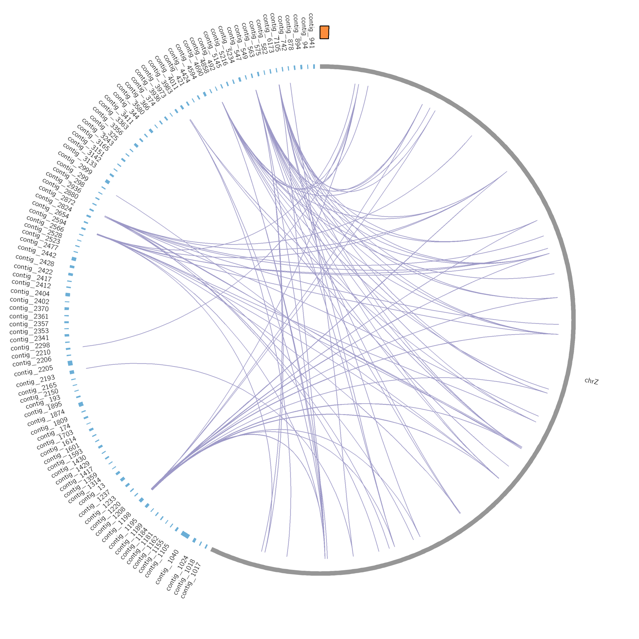

Supplement: S18 Fig — Each boxplot represents the f:m coverage ratio within the coordinates of each gene. Boxplot colors represent three dosage compensated states: compensated, partially compensated and not compensated. Solid horizontal line marks f:m = 1 and dashed horizontal line marks f:m = 0.5. Red vertical dashed line shows the PAR limit. (TIFF) [file pgen.1010901.s018.tiff]

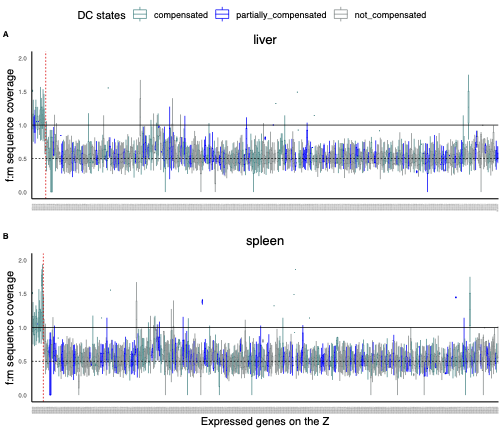

Supplement: S19 Fig — Contigs in blue represent the W chromosome and in gray the Z. The PAR in the Z chromosome is indicated in orange. Purple lines indicate matching regions between the W and the Z chromosome. (TIFF) [file pgen.1010901.s019.tiff]

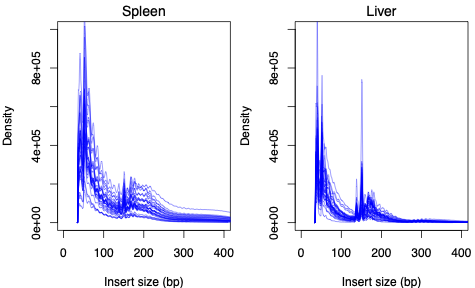

Supplement: S20 Fig — ATAC-seq insert size distribution for all biological replicates for spleen (left) and liver (right). The position of the first nucleosome can be observed between ~150–250 bp. (TIFF) [file pgen.1010901.s020.tiff]

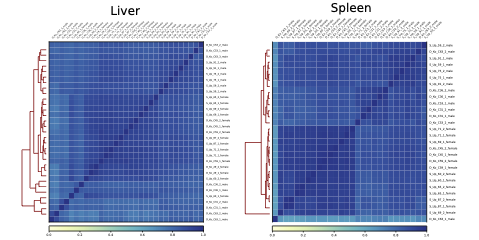

Supplement: S21 Fig — (TIFF) [file pgen.1010901.s021.tiff]

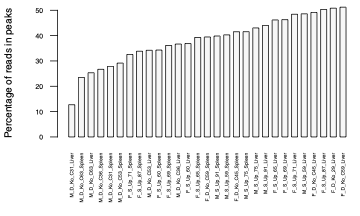

Supplement: S22 Fig — (TIFF) [file pgen.1010901.s022.tiff]

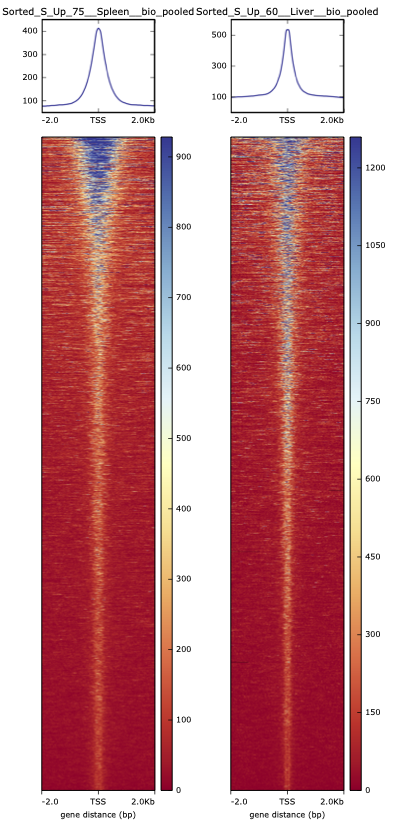

Supplement: S23 Fig — Upper panels: summary of coverage density +/- 2kb of the TSS, in spleen (left) and liver (right). Bottom panels: Enrichment of mapped reads presented as heatmaps along the defined TSS, in spleen (left) and liver (liver). (TIFF) [file pgen.1010901.s023.tiff]
