# Supplementary material for: Chromatin accessibility, not 5mC methylation covaries with partial dosage compensation in crows
Source: PLoS Genet. 2023 Sep 25;19(9):e1010901. doi: 10.1371/journal.pgen.1010901 (PMC10575545; doi:10.1371/journal.pgen.1010901)
Supplement: S1 Text — (DOCX) [file pgen.1010901.s024.docx]

**S1 Text**

**Identification of the pseudo-autosomal region (PAR)**

We used four independent lines of evidence to identify the PAR in crows.

1. *DNA sequencing coverage*

Along the female Z chromosome, we expected the PAR to have a female:male sequencing coverage close to 1, since the PAR has two copies in both sexes. Outside of the PAR, the genome coverage should be half in females, representing its haploid state. F:m coverage dropped at 688kb from a ratio of close to 1 to ~0.5 (**Figure 1, SA Fig**).

The possibility remains that regions in the Z outside of the PAR still show high homology to the W or that part of the PAR was assembled into the non-PAR region. In those cases, such regions would appear diploid and we would expect these to be dosage compensated. To check for such cases, we used the gametolog data set identified in neoaves and in the flycatcher and identified these in the Z of the European crow. The regions of our Z chromosome matching the gametologs in neoaves (**SB Fig**) and in the flycatcher (**SC Fig, SA Table**) showed a f:m genome coverage < 1, with one exception. The gene MSTRG.22228 showed a f:m coverage > 1, but interestingly this gene was not dosage compensated. Furthermore, we checked the f:m Z coverage within each expressed gene in liver and spleen and found that all of the genes outside of the PAR had a f:m DNA coverage < 1, with the exception of 6 genes in liver and 7 in spleen. Interestingly, not all genes outside of the PAR with a f:m genome coverage > 1 were fully dosage compensated (**SD Fig**).

1. *Heterozygosity levels*

We calculated heterozygosity and expected that the female PAR should harbor heterozygous sites, whereas the haploid part of the female’s Z, should be homozygous throughout. Males, on the contrary should show heterozygosity across the entire Z chromosome. Heterozygosity data was consistent with this expectation and identified the same boundary for the PAR as the coverage-based approach (**Figure 2)**.

1. *Orthology*

Avian karyotypes are conserved (Ellegren, 2010). We may thus expect to find similarity of the PAR to other passerine species. Strong homology with the already identified PAR in the flycatcher thus supports the identity of the PAR identified by the above means (**Figure 2**).

1. *Genome assembly*

While several contigs of the W assembly aligned to genes on the Z chromosome (gametologs) there were no hits detected for the candidate PAR region (**SE Fig**). This is consistent with the expectation that the PAR region is highly similar between the W and Z (as expected by recombination) and therefore has not been identified as a separate, divergent contig in the W assembly. The same holds true when comparing the W and Z chromosomes of the closely related New Caledonian crow for which a better W chromosome (VGP standard) assembly exists.
