# Supplementary material for: Chromatin accessibility, not 5mC methylation covaries with partial dosage compensation in crows
Source: PLoS Genet. 2023 Sep 25;19(9):e1010901. doi: 10.1371/journal.pgen.1010901 (PMC10575545; doi:10.1371/journal.pgen.1010901)
Supplement: S2 Text — (DOCX) [file pgen.1010901.s025.docx]

**S2 Text**

**Pre-processing of ATAC-seq data**

Quality control of ATAC-seq data was performed as described in the main document. Here, we provide details for each of the components. For each library we first examined the insert size distribution which should show a downward laddering pattern reflecting the amount and length of DNA fragments from nucleosome-free region and from regions with associated nucleosomes (Buenrostro et al., 2013). For all libraries, we observed an enrichment of fragments corresponding to the nucleosome-free region that ranged in length from 36 – 130 bp. The second enriched fragment observed, ranged from 150 - 250 bp in both organs, which corresponds to the mononucleosome-bound region (**SF Fig**). The detection of a mononucleosome ensures the boundaries for a successful detection of nucleosome-free regions, where putatively, the regulatory regions lie.

Next, we quantified the correlation between our two technical and biological replicates (8 females, 7 males for liver and spleen). For the technical replicates, Spearman correlation coefficients drawn from ATAC-seq coverage estimates ranged from 0.86 – 0.97 for liver and 0.72 – 0.98 for spleen (**SG Fig**). For biological replicates, Spearman correlation coefficients ranged from 0.87 to 0.95, where correlation was higher within the same sex for both tissues. The number of total usable reads (see methods), varied among samples (S**B Table**). Following the ENCODE guidelines for ATAC-seq quality, 50 million fragments are recommended for paired-end experiments. Due to a high correlation between technical replicates, we pooled these for downstream analysis, resulting in ~60 million reads for the smallest biological sample in liver and ~92 million reads in spleen.

We also revised the FRiP (Fragments of Reads in Peaks) score values for each pooled sample, which ranged from ~12 – 51% (S**H Fig**) and which evaluates the number of reads that were mapped into peaks. According the ENCODE guidelines, FRiP values exceeding 0.2 are acceptable. From our sample assessment, only one sample fell below this threshold which was accordingly removed from all subsequent analyses.

We further checked for an enrichment of mapped reads in putative transcription start sites (TSS) of the expressed genes which we defined as genes with FPKM expression levels exceeding 1. The putative TSS was identified as 2kb upstream and downstream of the first nucleotide of the expressed gene. Wig files were produced for each sample and heatmaps showing coverage enrichment around the TSS were done with Deeptools v.3.5.0 (deepTools -computeMatrix -b 2000 -a 2000) (Ramírez et al., 2016). S**I Fig**, shows two examples, one of liver and one of spleen, of coverage enrichment around the TSS (+/- 2kb).
