# Supplementary material for: Chromatin accessibility, not 5mC methylation covaries with partial dosage compensation in crows
Source: PLoS Genet. 2023 Sep 25;19(9):e1010901. doi: 10.1371/journal.pgen.1010901 (PMC10575545; doi:10.1371/journal.pgen.1010901)
Supplement: Attachment — Submitted filename: AC_JW_230616_Response_PG_P1_JW.docx [file pgen.1010901.s040.docx]

Dear Dr Catalán,

Thank you very much for submitting your Research Article entitled 'Epigenetic mechanisms of partial dosage compensation in an avian, female heterogametic system' to PLOS Genetics.

The manuscript was fully evaluated at the editorial level and by independent peer reviewers. The reviewers appreciated the attention to an important topic but identified some concerns that we ask you address in a revised manuscript.

We therefore ask you to modify the manuscript according to the review recommendations. Your revisions should address the specific points made by each reviewer. Please also ensure that your genomic data are made available before resubmitting your revised manuscript.

Dear Editor,

we are glad to hear that you find merit in our work and would like to thank you for the granted extension to conduct the revisions. Below, we expand on the specific points raised by each referee. In the revised version, we have also added a data availability statement specifying where all sequencing data and proprietary code can be found.

We thank you for considering publication of our work in PlosGenetics.

Your sincerely,

The authors.

**Reviewer #1**:

The paper shows the potential of combining gene expression data (RNA-Sequencing) and genome accessibility data (ATAC-Sequencing) in a non-model organism. In general, I think the experimental design and the analysis are of a very good standard. Even though it was already known that incomplete dosage compensation exists in crows, the paper nicely demonstrated how dosage compensation mechanisms reflects on chromatin level changes. Ultimately, however, the experimental design chosen here do not reveal the underlying molecular mechanism, but only a correlation between chromatin accessibility and gene expression. This the authors even admit themselves (e.g. in the text “We observed that gene expression covaried with peak height (Figure 4A) and observed that the highest peak is more often found in promoter regions than other parts of the gene or intergenic regions (Figure 4B).”) It is therefore important that abstract and text will be rephrased accordingly.
Otherwise, I think the paper makes a good case for publication in PLOS Genetics, if the points below are addressed. Lastly, I want to congratulate the authors on the methods paragraph, I think it has been really carefully written and contains many important details.

We thank the referee for their time and the many helpful and constructive points of feed-back. To avoid causal reasoning, we have now changed the title to ‘Chromatin accessibility, not 5mC methylation covaries with partial dosage compensation in crows’ and also modified the abstract accordingly.

Specific comments:

• The authors suggest a chromosome-wide epigenetic mechanism on the female Z-chromosome in female crows. However, it was also shown that the dosage compensation/dosage balance is incomplete and the majority of genes are unaffected. For example, the authors write that “Using a sliding window analysis employing windows of different sizes (PAR equivalents of 688 kb or 1 Mb) no significant clustering of compensated genes was found …” – wouldn’t this rather speak for a gene-by-gene manner rather than a chromosome-wide upregulation? The authors should explain and address how the observed upregulation in a gene-by-gene manner and a chromosome-wide epigenetic mechanism are compatible.

Both options are now discussed in more detail (lines: 455 - 465) This observation is compatible with a targeted gene-by-gene mechanism as previously been suggested for birds (Mank, 2009; Mank and Ellegren, 2009; Warnefors et al., 2017), or an overall more accessible chromosome-wide environment affecting upregulation of a subset of genes. Since increased chromatin accessibility was enriched, but not restricted to regulatory regions of female Z-linked genes, we lean towards a model of chromosome-wide regulation that is further modified on a gene by gene basis. Comparative evolutionary analyses between species are needed to disentangle the order of events.

I would like to have a bit more information about the genes which are affected by the DC/DB mechanism, or even a little follow up on those.

• What kind of genes are affected? Are the Genes suspected to be dosage sensitive like for example genes that code for proteins that are subunits of bigger complexes? Maybe one could even do a GO analysis or compare with data from different species (from other studies).

• Are the affected genes overlapping in both tissues?

On the Z chromosome, 45% and 43% of the genes are dosage balanced in liver and spleen respectively. From all of the dosage-balanced genes, 30.3% (194 genes) are shared between the two tissues. Motivated by the referee’s comment we have conducted a gene ontology analysis for dosage balance using the following sets of genes: (1) all dosage-balanced genes in liver OR spleen (2) dosage-balanced genes in liver AND spleen, (3) all dosage-unbalanced genes in liver OR spleen (4) dosage-unbalanced genes in liver AND spleen. From these categories, only the shared balanced genes between tissues yielded five significant GO terms. Interestingly as the reviewer suggested, the significantly GO categories involve protein complexes (e.g. PIK3R1, RICTOR), cell signaling (e.g. IQGAP2) and immune reaction (e.g. PTGER4), interactive functions where concentration of gene products might be essential to function optimally both in males and females. These results are mentioned in lines 240 - 245 and **Table S4**. We abstained from analyses of dosage compensated genes, since cross-chromosomal comparisons are likely invoking other Z vs. A factors unrelated to dosage effects.

• Is there any correlation between status of compensation and expression level?

We have done a correlation analysis between f:m expression ratios on the Z with gene expression levels measured as FPKM. We observe a negative correlation between dosage balance status and gene expression. The lower the expression level the more likely is that the genes is dosage balanced. This result would hint at a scenario where lower expressed genes are more sensitive to expression deviations between females and males. This result is mentioned in the lines lines 240 – 245.

**Figure 1**. Correlation analysis between dosage balance state as measured by log_2_(f:m) along the Z chromosome and log_2_(FPKM) mean gene expression.

I think it is great that the study takes different tissues into account. The results show differences between the tissues (Spleen/Liver) in terms of DC/DB and 5mC-Methylation. I think these differences could be addressed in more depth.

• Are the same Genes affected (see 2.)?

We identified 194 genes that are dosage compensated in both tissues (please see response above).

• Could it be a concern that liver cells are often polyploid? (or is this not the case in birds?)

According to the literature, polyploid cells in liver have only been identified in mammals. We therefore did not consider this as a concern.

Please see the following references:

<https://www.ncbi.nlm.nih.gov/pmc/articles/PMC6592246/>

<https://www.ncbi.nlm.nih.gov/pmc/articles/PMC8534039/>

<https://www.sciencedirect.com/science/article/pii/S000294401300655X>

• Should we conclude from the results that future studies should look at more tissues to get a more complete picture of the mechanisms in the organism?

This is certainly encouraged. However, while we found a sex-chromosome-by-tissue interaction for 5mC methylation, we could not disclose any tissue-specific effect on dosage balance or compensation, neither for the ATAC-seq nor 5mC data. We therefore chose not to highlight this aspect in the discussion.

DNA methylation data
• Could the authors briefly explain in the text the state-of-knowledge about DNA methylation in birds? Does it occur at the same sites as in mammals? The analysis implies that CpG islands also exists in birds, but can this perhaps be shown? Is there any evidence that DNA methylation is repressive in birds?

Indeed, as in most vertebrates, 5mC DNA methylation is a regulatory mark largely indicative of heterochromatin or repressed gene expression, when found near CpG islands within a gene promoter (Fujita *et al.,* 2003 – Journal of Biological Chemistry for CpG islands). We have now provided a summary sentence within the introduction referencing a study on the great tit in which they analyze multiple tissues and specifically examine CpG island methylation (Laine *et al.* 2016 – Nature Communications) (lines 117-120). While DNA methylation is also known in birds to be a repressive mark, the correlations with gene expression are not always straightforward. Few groups have specifically measured methylation and expression in common garden conditions (Lindner *et al.,* 2021 – BMC Genomics). We mention this work (lines 115 - 120) and hope our study contributes basic data on the topic.

• Since the Z chromosome is upregulated, I did not get the concept of why one would study a repressive mark per se? If the idea is that there is less methylation than in the ancestral state – is there a way that the authors could use DNA methylation data from an outgroup (chicken (?)) and ask whether the ancestral DNA methylation levels have been “erased” in crows? I don’t think this is a critical point, but perhaps (if the suggested analysis is not possible), the interpretations could be a bit toned-down.

The idea is indeed that the evolutionary pressure for upregulating the female Z stems from the need to equilibrate expression with interacting autosomal genes. Reducing methylation on the female Z may be one mechanism to achieve this. A comparative approach to clarify the evolutionary trajectory would indeed very valuable. However, evolution of the sex-limited female chromosome commenced likely at the base of the avian clade with the oldest stratum of avian sex chromosomes being about as old as all Aves (all non-ratites). Hence investigating ancestry of methylation would require very distant outgroups and complex analyses identifying concordant orthologs with methylation data available for all species. Such careful investigation of ancestral methylation and changes along the phylogeny makes for an exciting study which is, however, outside the scope of this manuscript.

Text
• “epigenetic control mediated by differential chromatin accessibility and to a lesser extent 5mC methylation between the sexes” in the abstract is an overstatement of the findings – if genes are more or less expressed they will obviously show differences in accessibility and/or 5mC, but this does not demonstrate by any means a causality for dosage compensation and/or balance, but instead is a correlation. This sentence from the abstract is just an example, but the entire text has to be adjusted accordingly.

We have cautiously revised and edited the manuscript, including the abstract, to avoid overstatements. Since we find no clear evidence for a contribution of 5mC to DC or DB we rephrased this aspect in the abstract altogether. In avian systems there is still plenty to understand how chromatin accessibility and 5mC patterns influence gene expression, and most of the studies looking at these interactions do no find a perfect 1:1 interaction. We discuss this matter and hope that our study contributes valuable, basic information.

• The authors should eliminate the word “epigenetic” from their text, because the analyses do not by any means address the inheritance patterns of the observed marks. Likely, all the observed effects are purely genetic. I guess the authors mean “chromatin-based regulation” and should use this phrasing instead.

*Epigenetics* in the classical sense (*epigenesis* sensu Waddington), is a field of research concerned with the processes governing organismal development: the orchestration of predictable ontogenetic change from single-celled zygotes to fully fledged, multi-cellular composites (‘genotype-to-phenotype’). A main goal is to gain a detailed understanding of the regulatory molecular mechanisms involved in cellular differentiation and phenotypic variation more generally.

During the last decades, much progress has been made in understanding these underlying molecular mechanisms (*epigenetic mechanisms*) that control ‘the interaction of genes and their products […] which bring the phenotype into being’ (Waddington, 2008 p. 242). In the 21^st^ century chromatin-based regulation, such as methylation and histone modification, have thus become the centre of attention which lead to a narrow definition of the term, largely equating it with ‘chromatin modification’ [1–3]. This definition of ‘epigenetics’ is consistent with them ultimately being of genetic origin (sensu Waddington).

Importantly, this use of the term epigenetics is not to be confounded with ‘epigenetic inheritance’. Literature on ‘epigenetic inheritance' emerged in parallel and in addition to cellular, molecular mechanisms considers higher-level dimensions such as developmental and behavioral interactions between parents and offspring, niche construction, cultural transmission[9–11] or the microbiome[12]. This aspect is indeed not of interest here. We are simply interested in molecular mechanisms associated with the chromatin that may explain a phenotype (gene expression).

We could revert many of the ‘epigenetic’ instances in the text to ‘chromatin-based regulation’ as suggested by the referee. Yet, the term epigenetics is ubiquitously used in the classical sense mentioned above throughout the dosage compensation literature. Regulatory chromatin-based mechanisms are routinely referred to as ‘epigenetic mechanisms’, ‘epigenetic changes’, ‘epigenetic aspects of dosage compensation’, ‘epigenetic landscape’, ‘epigenetic assignment’, etc. [4–8]. We therefore chose to follow common practice and stick with the term. To avoid confusion, we removed the term from the title and define the way we use it here upfront.

• Intro: “For example, in mammals one of the X chromosomes is randomly targeted for inactivation in the homogametic sex by nucleosomal condensation” – not sure what is meant with “nucleosomal condensation” – please rephrase.

What was meant with nucleosomal condensation, is the presence of transcriptome arrest that one of the mammalian X chromosomes undergoes, as one of the steps, to equalize X chromosome expression. We have rephrased this sentence and changed “nucleosomal condensation” to “chromatin modifications” (lines 75 - 80). As mentioned in Brockdorff and Turner 2015, silencing of the X chromosome is achieved at the level of chromatin structure by histone modification, DNA methylation of CpG islands, and reorganization of chromatin folding.

• Intro: “The active X chromosome is then hyper-transcribed to equalize expression levels to the (diploid) autosomal dose (Di and Disteche, 2006).” Rephrase - This statement is not correct as such, the upregulation of the active X in mammals involves multiple layers, including transcript stability and translational regulation and its mechanism is not full resolved yet.

We edited this part of the introduction and added Lentini et al. 2022 as additional reference. We do not seek to go into much detail on mammalian dosage compensation, as the mechanism is complex, but revised the passage to ensure that the given information is correct (lines 75 - 80).

• Intro: “In heterogametic ZW systems, with the exception of several moth species (Huylmans et al., 2017; Smith et al., 2014), …” also other ZW systems show full compensation, e.g. brine shrimp Artemia franciscana, turtle Apalone spinifera or Schistoma mansoni (at least in some tissues). Please rephrase.

Thanks for reminding us about other examples of ZW systems with dosage compensation. We have rephrased this part and added the corresponding references (lines 90 - 95).

• The discussion is a bit lengthy, perhaps one could shorten it a bit.

We shortened the discussion in places and moved technical aspects to the material and methods section, and shortened where deemed appropriate (see e.g. section moved to methods section lines 860 - 870).

• Perhaps I missed it, but when did the Z chromosome of crows evolve? Is it the same Z as chicken (i.e. the chicken / crow Z evolved before the species split)? It would be helpful to add this info in the text.

See comment above. The sex chromosomes in birds are highly syntenic and it has been proposed that the Z chromosome of birds share a common ancestor that predates modern birds. This information has been added to the introduction (lines 95 - 100).

Figures:
• Figure1A: f:m ratio for the PAR region is above the expected value of 1 (almost 1.5). As the hemizygous parts are also a bit a bit over 0.5, there is likely to be technical explanation.

Thanks for pointing this out. We also wondered where the variation along the Z (incl. PAR) comes from. We performed this analysis on a masked genome to diminished noise coming from repeats. The f:m coverage was additionally normalized to autosomal values as explained in the methods section (lines 805 - 855). Still, we find substantial variation in coverage which we attribute to variation in mapping success, in particular in regions of (unmasked repeats). Larger window-sizes would alleviate this problem, but are not appropriate due to the fact that the PAR is so short.

• Figue1C: I am not quite sure if this graphic tells anything relevant for the understanding of the paper. It is also not mentioned in the text.

Thank you for pointing out that we missed referencing Figure 1C in the text. Figure 1C, shows our third line of evidenced for the identification of the PAR, which is based on homology to the flycatcher. It is now referenced in the text, lines 165 - 170.

**Bibliography**

1.	Wright AE, Zimmer F, Harrison PW, Mank JE. Conservation of Regional Variation in Sex-Specific Sex Chromosome Regulation. Genetics. 2015;201: 587–598. doi:10.1534/genetics.115.179234

2.	Xu L, Wa Sin SY, Grayson P, Edwards S V, Sackton TB. Evolutionary Dynamics of Sex Chromosomes of Paleognathous Birds. Genome Biology and Evolution. 2019;11: 2376–2390. doi:10.1093/gbe/evz154

3.	Jablonka E, Lamb MJ. The Changing Concept of Epigenetics. Annals of the New York Academy of Sciences. 2002;981: 82–96. doi:10.1111/j.1749-6632.2002.tb04913.x

4.	Deichmann U. Chromatin: Its history, current research, and the seminal researchers and their philosophy. Perspectives in Biology and Medicine. 2015;58: 143–164. doi:10.1353/pbm.2015.0024

5.	Deichmann U. Epigenetics: The origins and evolution of a fashionable topic. Developmental Biology. 2016;416: 249–254. doi:10.1016/j.ydbio.2016.06.005

6.	Pigliucci M. Do we need an extended evolutionary synthesis? Evolution. 2007;61: 2743–2749. doi:10.1111/j.1558-5646.2007.00246.x

7.	Laland KN, Uller T, Feldman MW, Sterelny K, Müller GB, Moczek A, et al. The extended evolutionary synthesis: its structure, assumptions and predictions. Proceedings of the Royal Society B: Biological Sciences. 2015;282: 20151019. doi:10.1098/rspb.2015.1019

8.	Ashe A, Colot V, Oldroyd BP. How does epigenetics influence the course of evolution? Philosophical Transactions of the Royal Society B: Biological Sciences. 2021;376: 20200111. doi:10.1098/rstb.2020.0111

9.	Angers B, Perez M, Menicucci T, Leung C. Sources of epigenetic variation and their applications in natural populations. Evolutionary Applications. 2020;13: 1262–1278. doi:10.1111/eva.12946

10.	Graves JAM. The epigenetic sole of sex and dosage compensation. Nat Genet. 2014;46: 215–217. doi:10.1038/ng.2903

11.	Park Y, Kuroda MI. Epigenetic Aspects of X-Chromosome Dosage Compensation. Science. 2001;293: 1083–1085. doi:10.1126/science.1063073

12.	Richard G, Legeai F, Prunier-Leterme N, Bretaudeau A, Tagu D, Jaquiéry J, et al. Dosage compensation and sex-specific epigenetic landscape of the X chromosome in the pea aphid. Epigenetics & Chromatin. 2017;10: 30. doi:10.1186/s13072-017-0137-1

13.	Sun D, Maney DL, Layman TS, Chatterjee P, Yi SV. Regional epigenetic differentiation of the Z Chromosome between sexes in a female heterogametic system. Genome Res. 2019;29: 1673–1684. doi:10.1101/gr.248641.119

14.	Samata M, Akhtar A. Dosage Compensation of the X Chromosome: A Complex Epigenetic Assignment Involving Chromatin Regulators and Long Noncoding RNAs. Annu Rev Biochem. 2018;87: 323–350. doi:10.1146/annurev-biochem-062917-011816

15.	Sun D, Maney DL, Layman TS, Chatterjee P, Yi S V. Regional epigenetic differentiation of the Z Chromosome between sexes in a female heterogametic system. Genome Research. 2019;29: 1673–1684. doi:10.1101/gr.248641.119

**Reviewer #2:**

This manuscript takes a detailed look into the mechanism of partial dosage compensation in crows, and shows evidence for a chromosome wide epigenetic regulation mechanism. This has been previously hypothesised, but not tested so this manuscript nicely fills this gap in knowledge. The manuscript is well written and has sufficient data to test the hypotheses. I have very few comments.

We were pleased to hear this (near-immaculate) positive feed-back.

The main one is that although the authors have produced and analysed a nice dataset the authors have not made the data nor their code available! – this should be done prior to submission following PLoS’ data policy.

We have now uploaded all data and code (https://github.com/EvoBioWolf/2023_CORVID_DosageCompensationBalance) and added a data availability statement. Please check Table S9 for SRA accession numbers.

Fig.2. Please use more contrasting colours – I find it hard to see the difference between the two blues.

We have changed the colors in the figure and hope this helps.

**Reviewer #3**:

Dosage compensation as a result of sex chromosome differentiation is achieved by different mechanisms and in the past has been predominately investigated at the transcriptional level (with the exception of human and mouse and to some extend chicken). The work is thorough, state of the art and does provide novel aspects on dosage compensation on the chromatin level that are important for this area of research. The data on DNA methylation and chromatin accessibility in relation to gene expression do provide a step forward in understanding how gene and chromosome organisation evolved during sex chromosome evolution and also provides important revision of the idea of region specific differential methylation in the ZZ/ZW system.

Overall I would encourage the authors to revise the manuscript so it is a bit easier for readers outside the specific area (including the title which I find a bit cryptic).

We have changed the title (see also comment of referee 1).

The authors could consider to just describe that sex chromosome differentiation and the associated gene loss from the heteromorphic sex chromosomes has led to various mechanisms to achieve dosage compensation. I personally find the framing around genetic sex determination a bit of a distraction this is fundamentally a result of sex chromosome differentiation.

We feel that having a very short introduction on sex chromosome evolution does help to put the topic of dosage compensation in perspective, but now have reduced this evolutionary aspect to a single sentence.

Abstract: Why is it relevant that the crows were raised under garden conditions?

We feel that this is an important point. Transcriptome profiles can show high variability due to different environmental factors and thus reduce experimental power. A common garden experiment is expected to minimize gene expression fluctuations due to variation in food, temperature, interspecific interactions or age. In case of birds much research is still conducted without environmental control. The mention in the abstract hopefully raises awareness to this experimental aspect.

Line 99: “trapped in an arrested stage” what does this mean? Undifferentiated sex chromosomes have been described in several species

We have re-written this passage (lines 70 - 80). In the former version of the manuscript what we meant as “arrested stage”, is the arrest or silencing of one of the X chromosomes in mammals, which takes place during the process of dosage compensation.

Lone 108-11, I find “the former” ,…, “the latter” always cumbersome and I suggest to define dosage balance and dosage compensation directly

We have replaced former and letter with the actual terms.

124: “in some species” has a MHM been show for any other species that chicken? This becomes clearer in the discussion.

We have clarified this part in the introduction and stated that MHM has been identify in birds belonging to the Galloanserae group (lines 115 – 125).

The authors could consider moving the description of the crow karyotype to the introduction.

We decided to keep the part where we describe the crow’s karyotype in the results section, as it interferes with the flow of the introduction which now focuses on dosage compensation and not chromosome evolution.

Figure 1C shows a comparison between crow and flycatcher. There is no mention in the results section about what has been observed.

Thank you for pointing out that we missed referencing Figure 1C in the text. It is now referenced in the text (lines 165 - 170).

Table 1 : I suggest to shorten the description in the table header and describe much of it in the actual results text.

We have shortened the header of **Table 1** and made sure that the information that is presented in this table is addressed in the text.

Line 373: “we found no orthologous region…” what does this mean? Did you look at the orthologous region of the chicken MHM? That should be spelled out.

Yes, we took the genomic region corresponding to the MHM in chicken and blasted it to the crow’s Z chromosome with the aim of finding an orthologous region in this species. This is described in the methods section (lines 790 - 800) and clarified in the results (lines 370 - 375).

Discussion: the discussion should be less technology heavy in the description of the results in my opinion. Avian dosage compensation has always been challenging to quantify and this paper does provide very sound data on 5mC and chromatin accessibility that shows no differential methylation (apart from spleen) but differential chromatin accessibility.

We have now moved the technological consideration of dealing with ATAC-seq data in the context of dosage differences to the methods section (lines 690 - 700).

Line 414: “see Chen et al.? Why?

We have removed this citation, as it is not vital for this point.

Lines: 425-440: although a bit of a side story I found these observations really interesting. Not sure how to better integrate in the manuscript.

This paragraph advocates the use of comprehensive statistical models in the study of dosage compensation accounting for confounding variables. We also thought about placing it more center-stage in the result section, but eventually abstained, as it disrupted the narrative and flow of the text. Its current position in the discussion may not be ideal, but we feel that the interested reader can still find it. To provide context for this statement, we now refer to the supplementary information displaying the effect sizes of the ‘confounding’ variable in this study.

Line 445: In the discussion I suggest to replace peak high by directly describing if chromatin was more accessible or not.

In most of the cases we have replaced peak height with chromatin accessibility, with the exception of the cases where it was necessary mentioning peak height as the trait to be analyzed in ATAC-seq data.

Line 447: the regions of differential accessibility are of interest but I feel they are not discussed very well, so these regions show a female bias in accessibility, what are the sequence features in this region, I take it that this is independent of 5mC but might be worth to spell out again.

The enrichment analysis of chromatin accessibility in the Z chromosome is independent of the 5mC analysis. For the 5mC analysis, an enrichment analysis was done as well, leading to no specific clustering or hypo- or hypermethylated sites on the Z. We have now added an enrichment analysis of the genes residing on the identified regions on the Z with female-biased chromatin accessibility but found no significant GO enrichment in either of the organs.

Line 461: “Throughout the manuscript”? Again this section makes an important point but it seems a bit disconnected.

We have now better embedded this section (lines 690 - 700).

It is great to see the numbers of individuals and looking at more than one tissue. Is there anything noteworthy in terms of individual differences in the assays, also why liver and spleen and do the authors have any idea about the differences they observed in spleen?

We chose to explore first only two organs to test the presence of a relation between ATAC-seq and 5mC patterns in dosage compensation. We chose liver and spleen as these organs do not present obvious sexual dimorphism, which could add another layer of complexity to this study.

In mammals the field has now moved to look at this from the proteom level and this may also be mentioned in this paper as a future direction in the avian clade.

This is a good point, and first examples also show in birds. We now mention in the discussion the usage of the proteome in investigating dosage compensation (lines 525 – 530).

<https://www.nature.com/articles/s41586-020-2899-z>

<https://www.ncbi.nlm.nih.gov/pmc/articles/PMC7652333/pdf/pgen.1009091.pdf>

https://academic.oup.com/mbe/article/32/10/2716/1211529

Overall a really thorough study by the authors with important insights into Z chromosomes expression between sexes.

We thank the referee for their time and are glad for the positive, constructive feed-back.
